# Supplementary figures and images for: Effect of set up protocols on the accuracy of alchemical free energy calculation over a set of ACK1 inhibitors
Source: PLoS One. 2019 Mar 12;14(3):e0213217. doi: 10.1371/journal.pone.0213217 (PMC6413950; doi:10.1371/journal.pone.0213217)

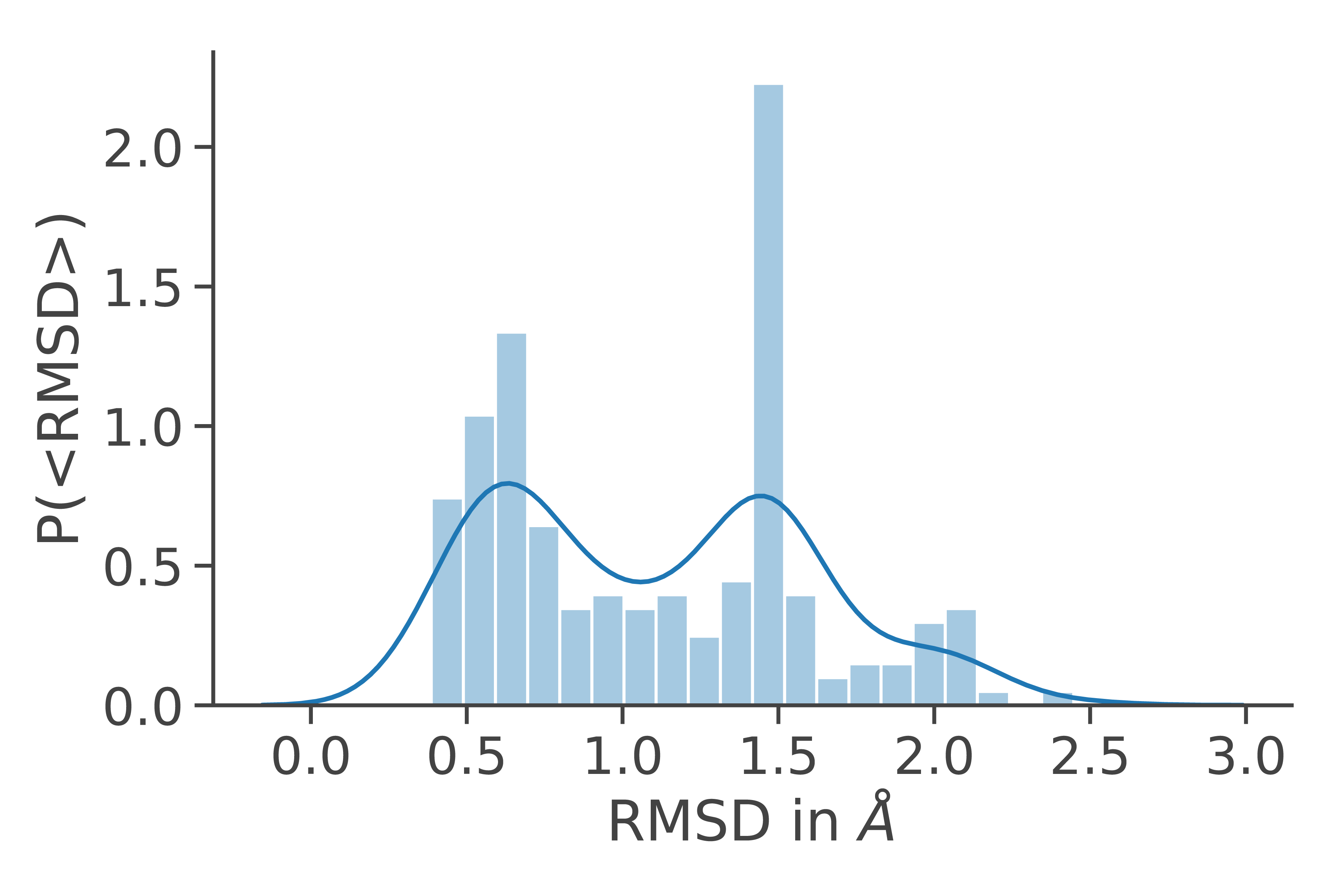

Supplement: S1 Fig — (TIF) [file pone.0213217.s001.tif]

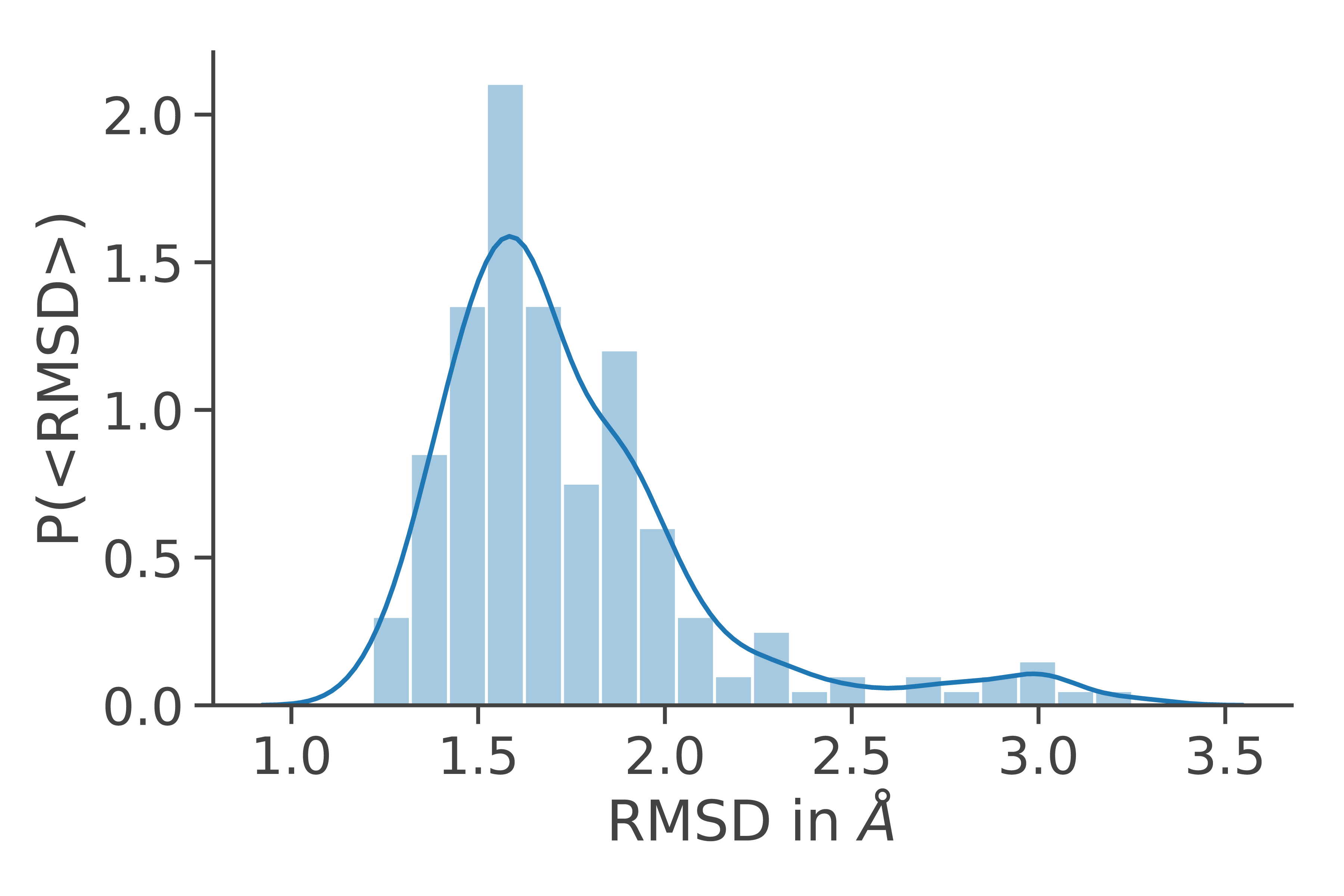

Supplement: S2 Fig — (TIF) [file pone.0213217.s002.tif]

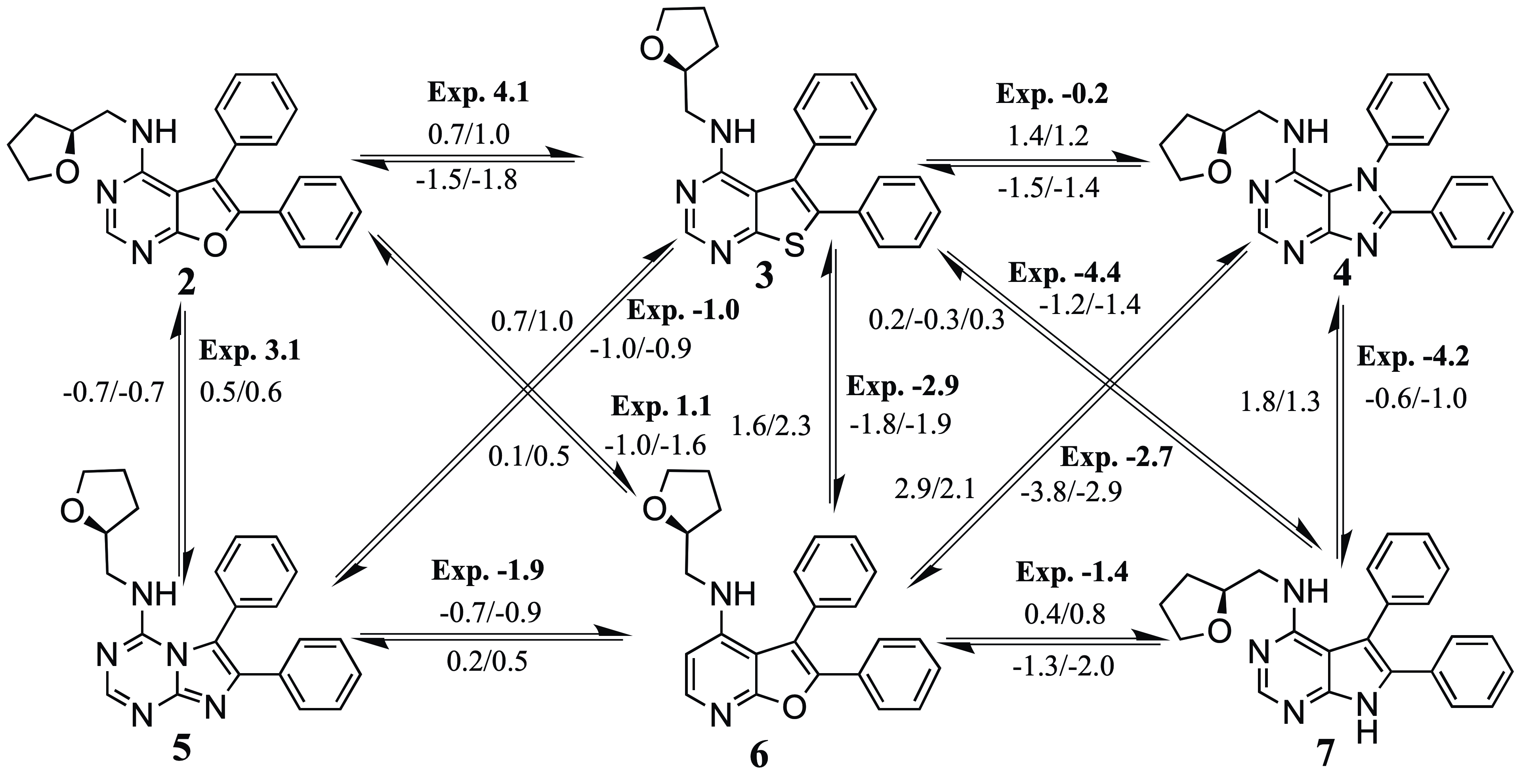

Supplement: S5 Fig — The drawing tries to reflect the different conformations adopted by ligands 2, 4 and 7. The calculated values correspond to independent repeats. (TIF) [file pone.0213217.s005.tif]

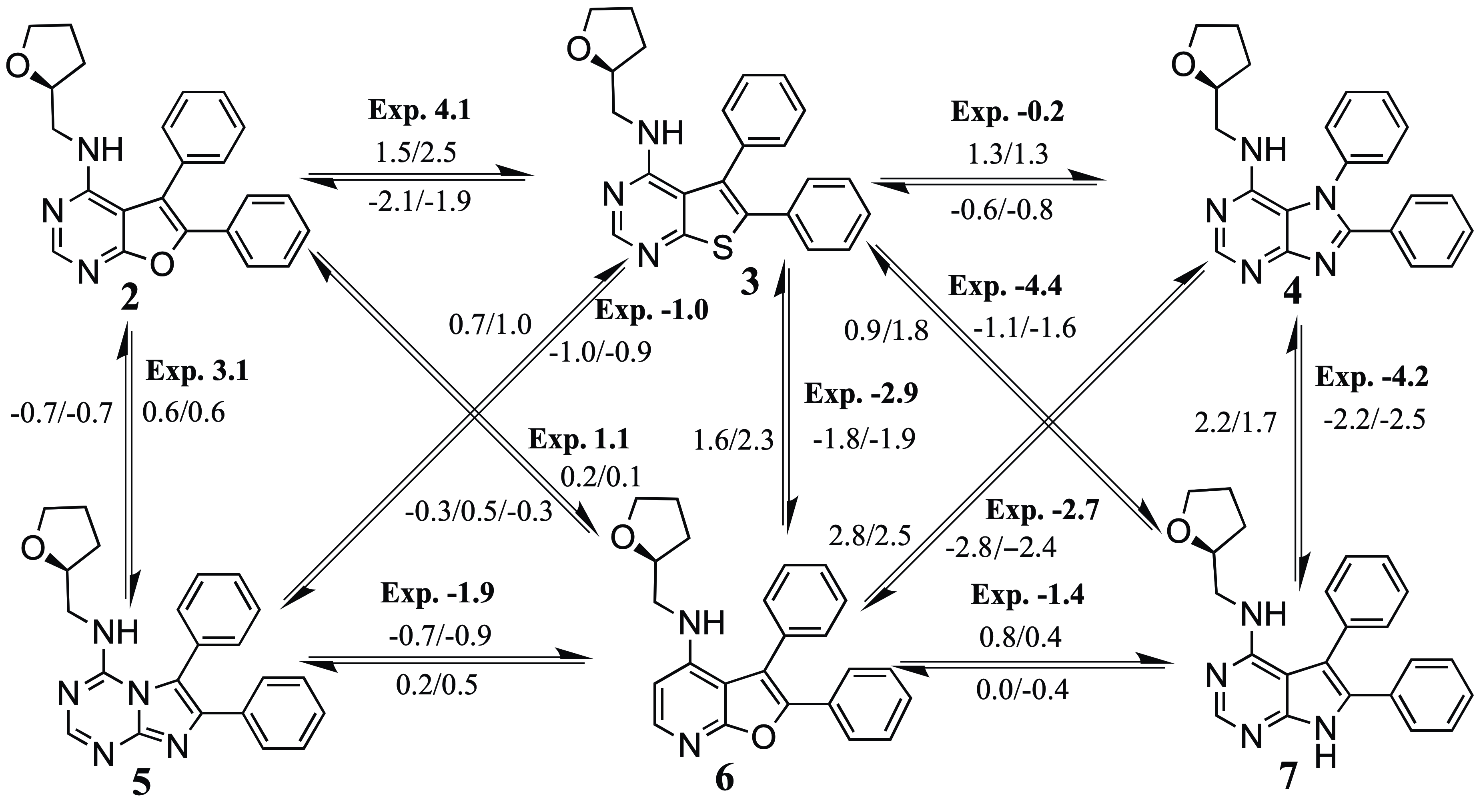

Supplement: S6 Fig — The calculated values correspond to independent repeats. (TIF) [file pone.0213217.s006.tif]

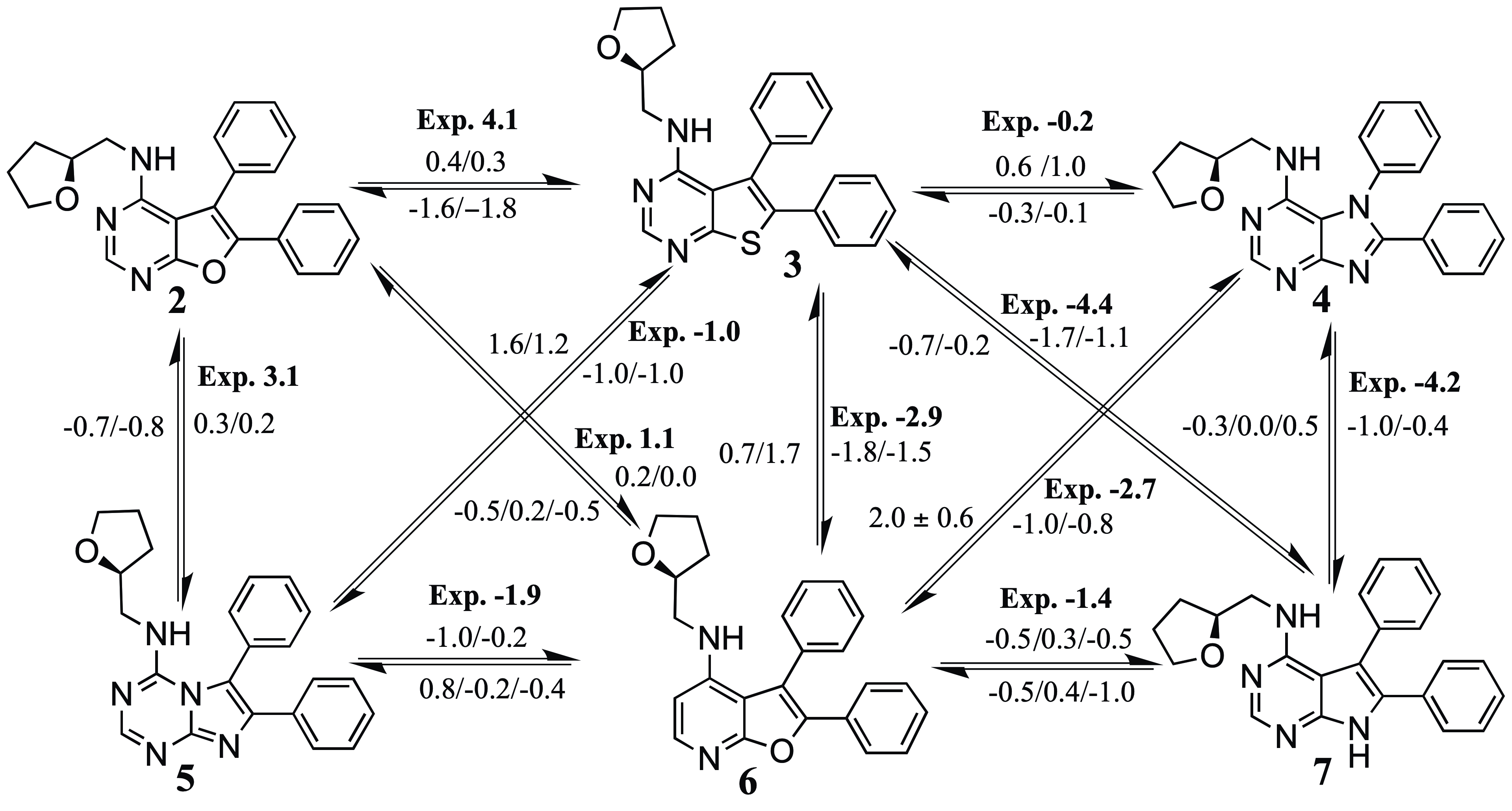

Supplement: S7 Fig — The drawing tries to reflect the different conformations adopted by ligands 2, 4 and 7. The calculated values correspond to independent repeats. (TIF) [file pone.0213217.s007.tif]

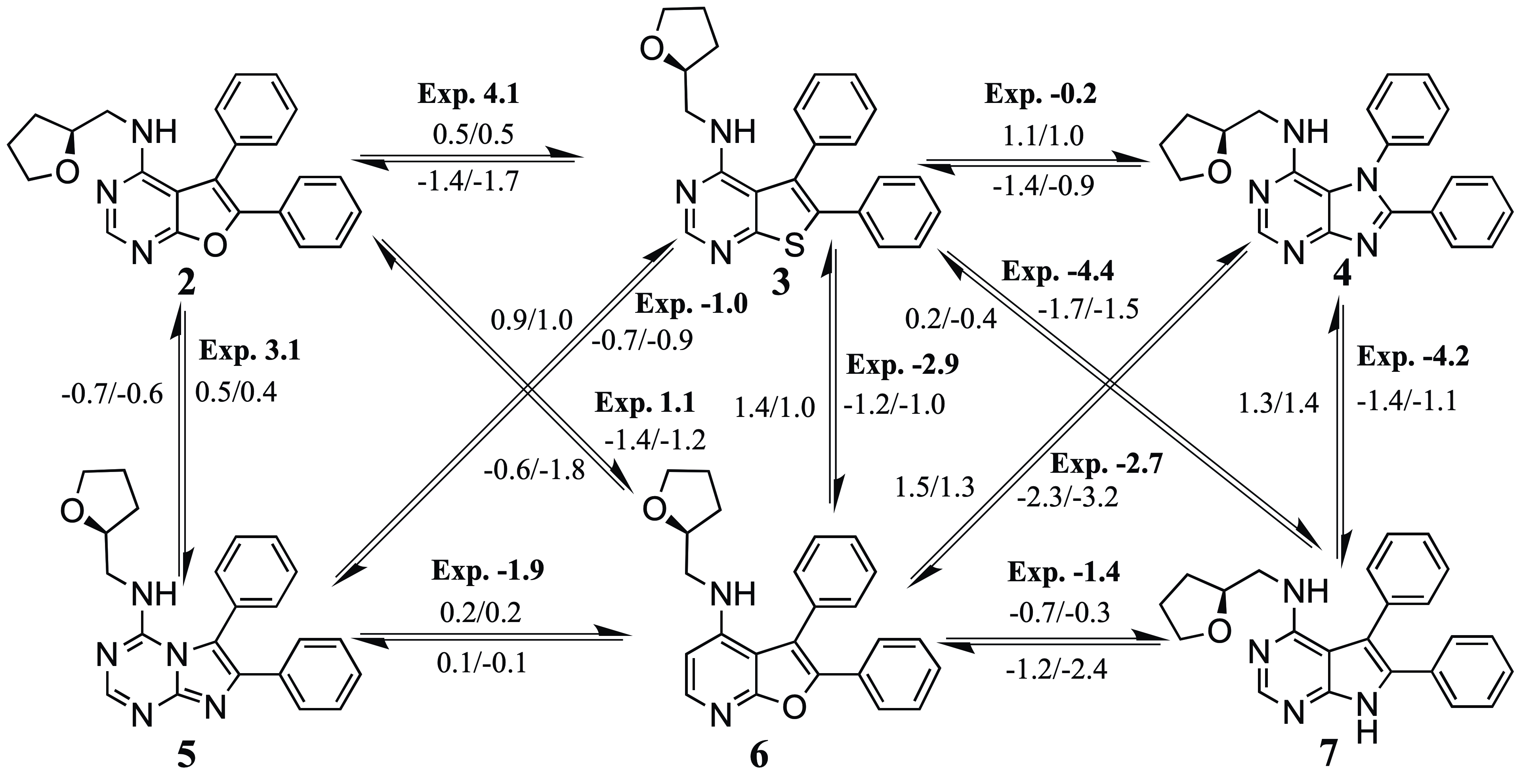

Supplement: S8 Fig — The drawing tries to reflect the different conformations adopted by ligands 2, 4 and 7. The calculated values correspond to independent repeats. (TIF) [file pone.0213217.s008.tif]

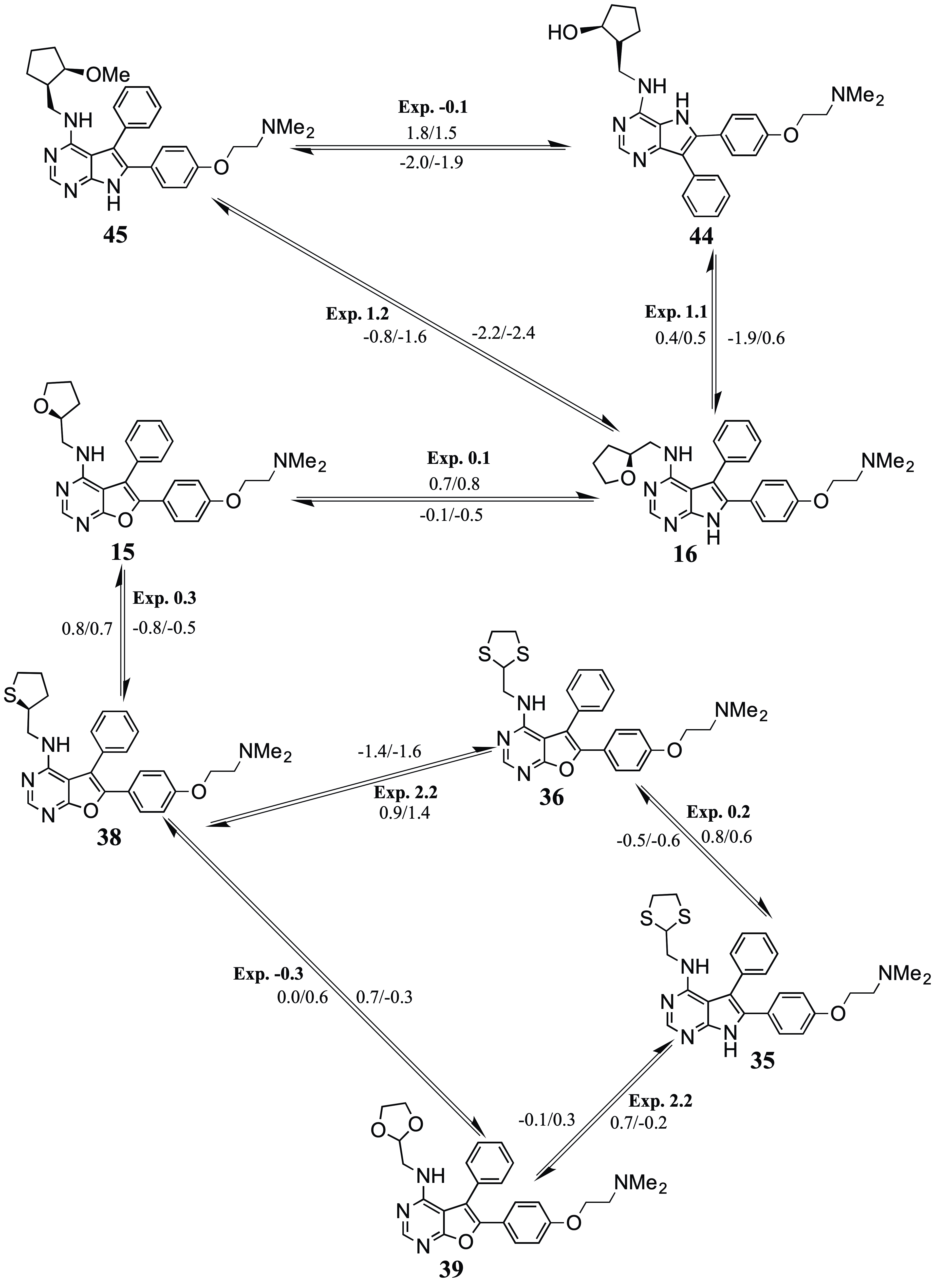

Supplement: S14 Fig — The drawing tries to reflect the different conformations adopted by ligands 44 and 16. The calculated values correspond to independent repeats. (TIF) [file pone.0213217.s014.tif]

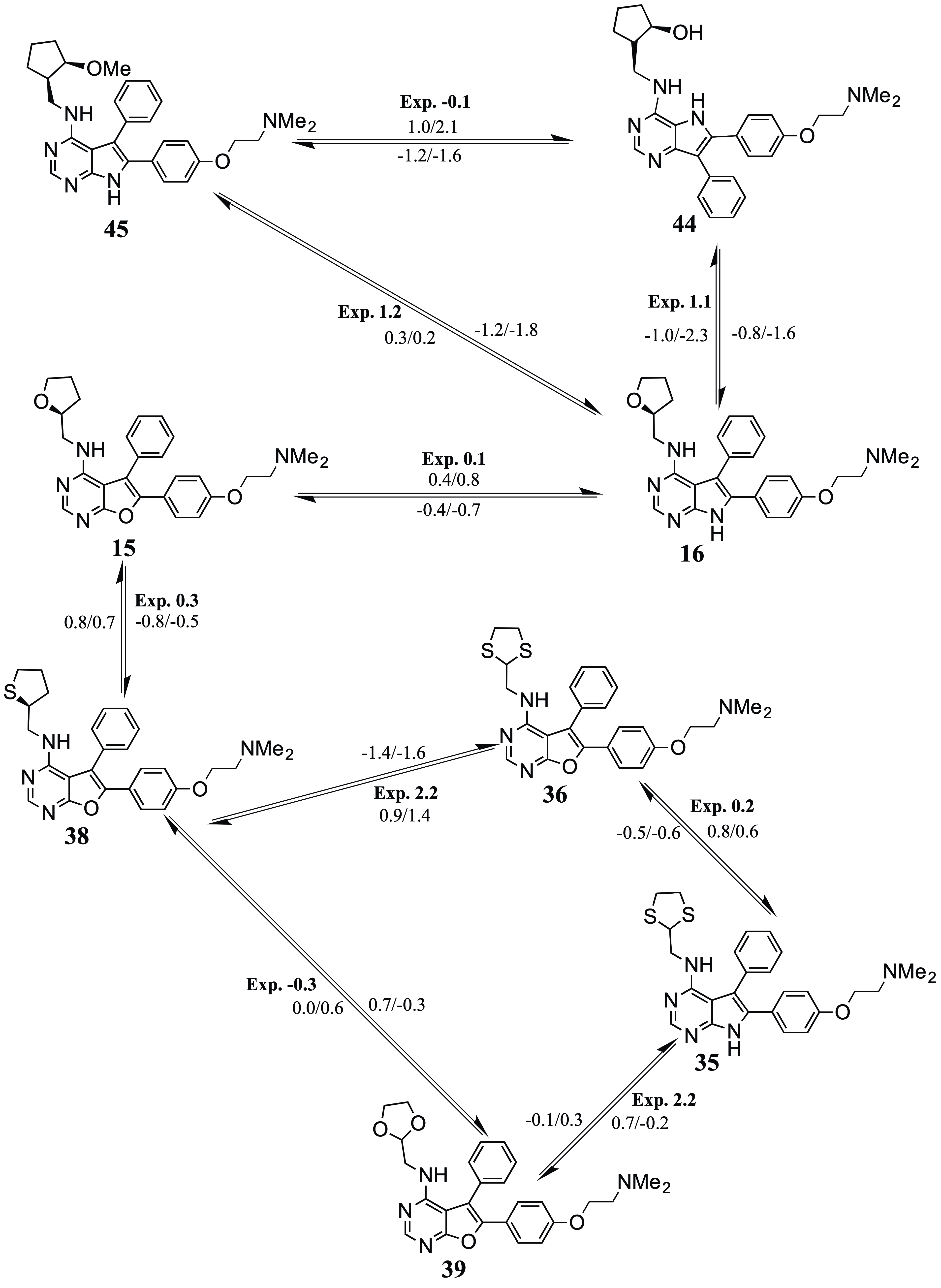

Supplement: S15 Fig — The calculated values correspond to independent repeats. (TIF) [file pone.0213217.s015.tif]

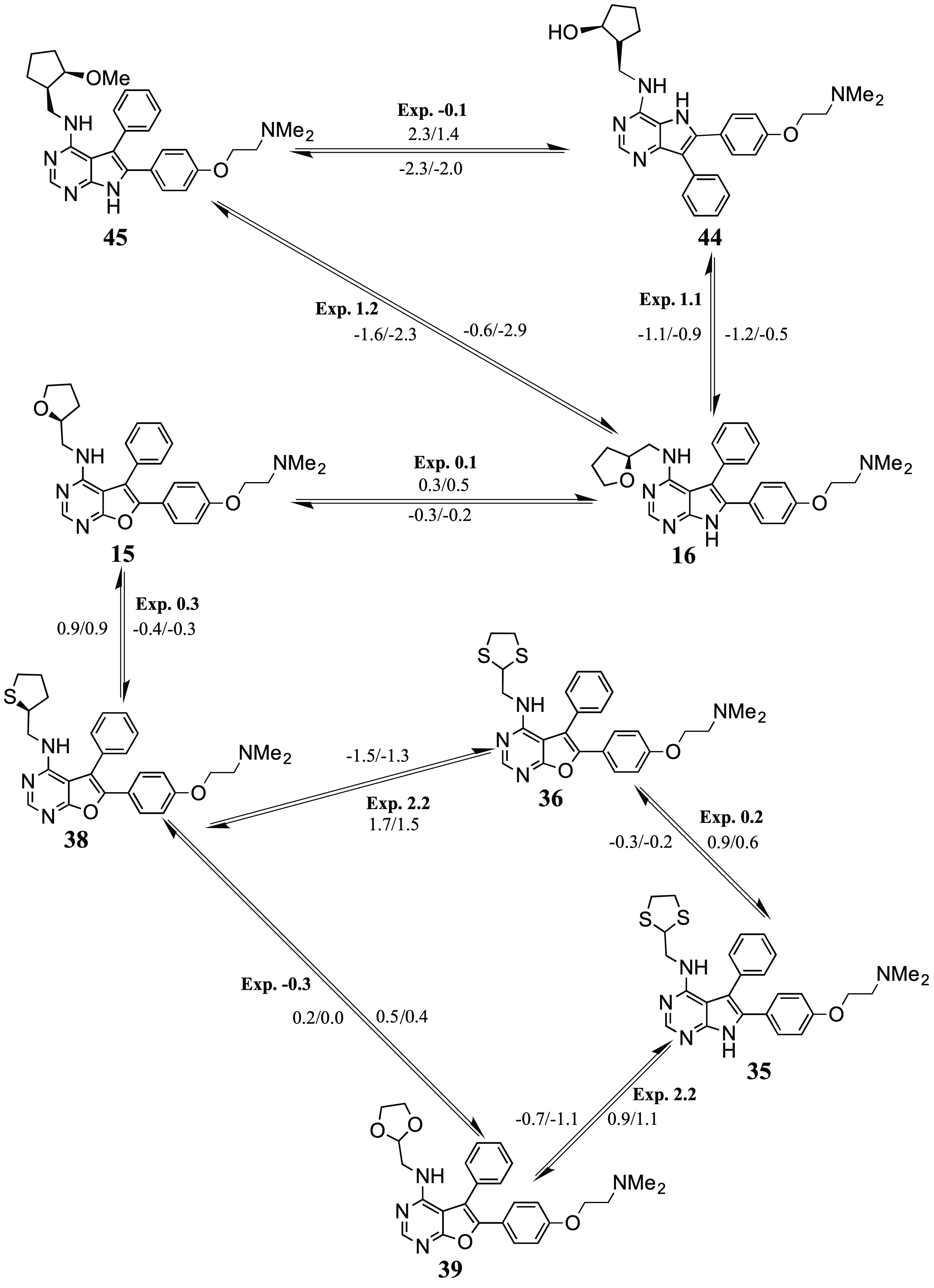

Supplement: S16 Fig — The drawing tries to reflect the different conformations adopted by ligands 44 and 16. The calculated values correspond to independent repeats. (TIF) [file pone.0213217.s016.tif]

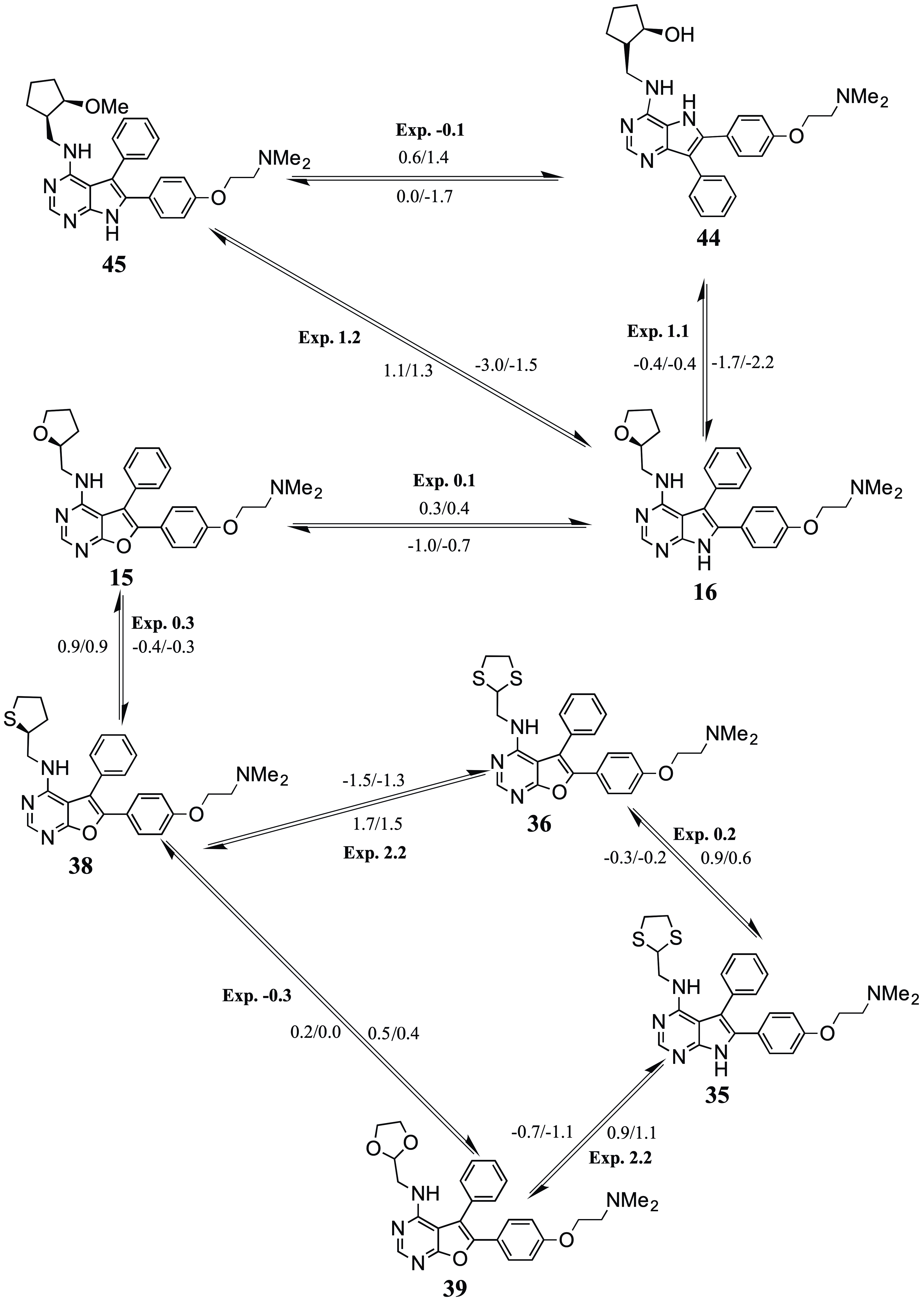

Supplement: S17 Fig — The calculated values correspond to independent repeats. (TIF) [file pone.0213217.s017.tif]

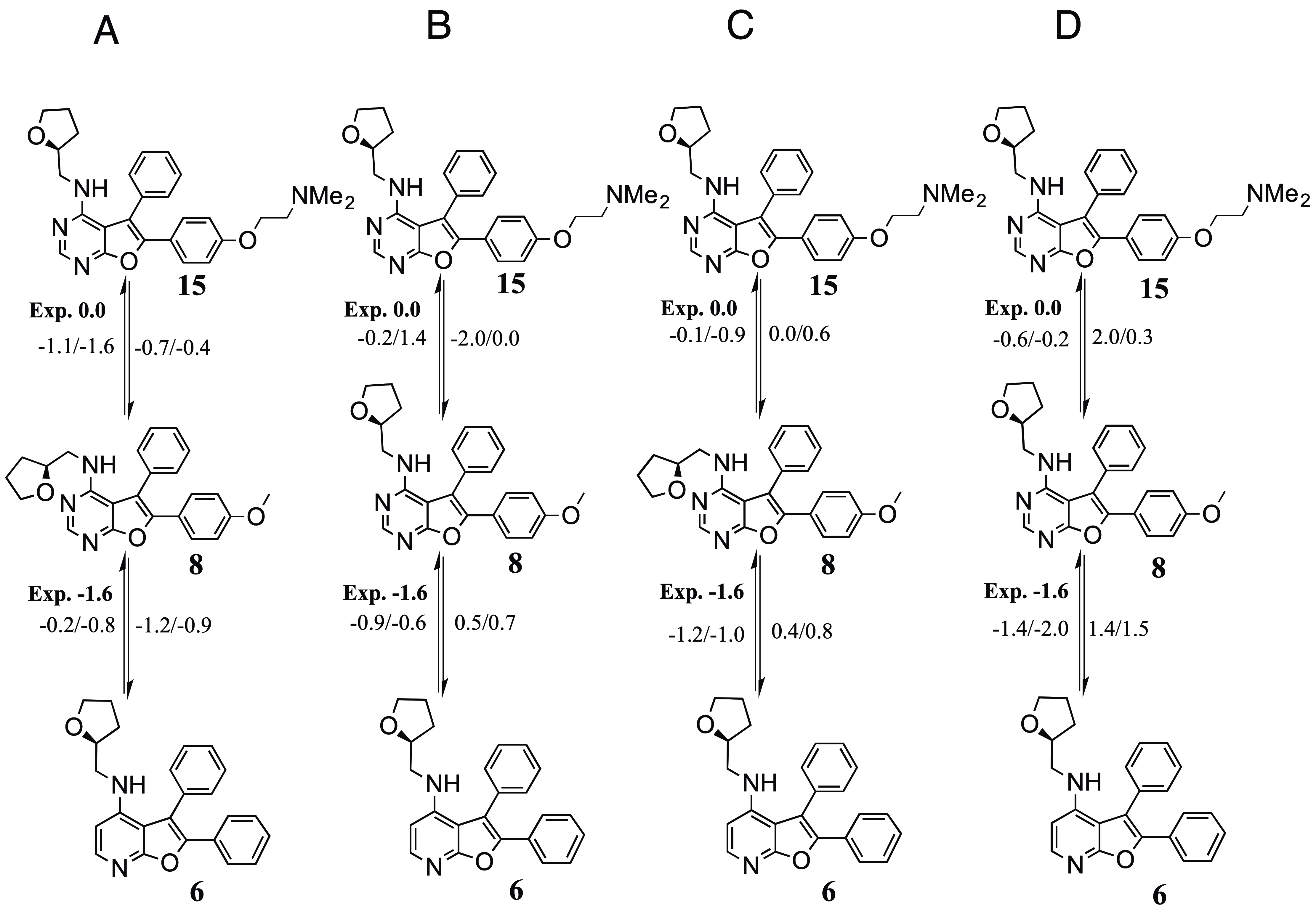

Supplement: S18 Fig — The calculated values correspond to independent repeats. (TIF) [file pone.0213217.s018.tif]
